# The cellular microenvironment and cytoskeletal actin dynamics in liver fibrogenesis

**DOI:** 10.32604/biocell.2022.020171

**Published:** 2022-05-18

**Authors:** Nour HIJAZI, Don C. ROCKEY, Zengdun SHI

**Affiliations:** Medical University of South Carolina, Charleston, 29425, USA

**Keywords:** Extracellular matrix, Actin dynamics, Transcriptional regulation, Signaling

## Abstract

Hepatic stellate cells (HSCs) are the primary effector cells in liver fibrosis. In the normal liver, HSCs serve as the primary vitamin A storage cells in the body and retain a “quiescent” phenotype. However, after liver injury, they transdifferentiate to an “activated” myofibroblast-like phenotype, which is associated with dramatic upregulation of smooth muscle specific actin and extracellular matrix proteins. The result is a fibrotic, stiff, and dysfunctional liver. Therefore, understanding the molecular mechanisms that govern HSC function is essential for the development of anti-fibrotic medications. The actin cytoskeleton has emerged as a key component of the fibrogenic response in wound healing. Recent data indicate that the cytoskeleton receives signals from the cellular microenvironment and translates them to cellular function—in particular, increased type I collagen expression. Dynamic in nature, the actin cytoskeleton continuously polymerizes and depolymerizes in response to changes in the cellular microenvironment. In this viewpoint, we discuss the recent developments underlying cytoskeletal actin dynamics in liver fibrosis, including how the cellular microenvironment affects HSC function and the molecular mechanisms that regulate the actin-induced increase in collagen expression typical of activated HSCs.

## Introduction

The cellular microenvironment—consisting of extracellular matrix, cells, and interstitial fluid (Warrick et al., 2008)—mediates essential cellular activities ranging from survival, growth, and motility/migration to gene regulation (Bloom and Zaman, 2014; Cheng et al., 2021). The extracellular matrix (ECM) not only provides physical support for cells, but also transmits both biophysical and biochemical signals to regulate intracellular activities (Clause and Barker, 2013; Frantz et al., 2010) through transmembrane receptors such as integrins to focal adhesion complexes to the actin cytoskeleton (Heng and Koh, 2010; Lee and Dominguez, 2010). The actin cytoskeleton is a highly dynamic structure, which by virtue of polymerization and depolymerization, works as a platform to convert biophysical forces into biochemical signals (and vice versa) to regulate downstream signaling pathways, a process known as mechanotransduction (Blanchoin et al., 2014; Gardel et al., 2010; Martino et al., 2018). Although it has been established that actin dynamics translate microenvironmental cues to cellular functions, the molecular mechanisms underlying this interplay are complex and remain to be fully elucidated. Recent studies from our group and others have shown that manipulating cytoskeletal actin dynamics or altering actin isoform composition exerts prominent effects on type I collagen expression, the major ECM protein in various types of fibrotic diseases. In this viewpoint, we focus on recent developments underlying cytoskeletal actin dynamics in liver fibrosis.

## Main Text

### The cellular microenvironment modulates cell phenotype

In normal liver, hepatic stellate cells (HSCs) reside in the subendothelial space, between the basolateral surface of hepatocytes and the anti-luminal side of sinusoidal endothelial cells, where the normal ECM consists largely of fibronectin, laminin, and minor quantities of types 1, III, IV, V, and VI collagen (Bedossa and Paradis, 2003; Martinez-Hernandez and Amenta, 1993). Under normal conditions, HSCs are in a quiescent state characterized largely by their abundant vitamin A lipid droplets (Jophlin et al., 2018; Shi et al., 2020). Upon liver injury (caused by viral infection, ethanol, and others), HSCs are exposed to proinflammatory substances secreted from nearby damaged hepatocytes, endothelial cells and immune cells, such as DAMPs (damaged-associated molecular patterns), interleukins, and growth factors. HSCs subsequently undergo a process termed “activation” where they transdifferentiate into myofibroblast-like cells with proliferative, migratory, contractile, and matrix-producing capabilities (Kisseleva and Brenner, 2021; Rockey, 2013; Tsuchida and Friedman, 2017). Additionally, activated HSCs secrete multiple pro-fibrotic growth factors such as TGFβ and ET-1, creating autocrine-stimulatory loops that further drive their myofibroblast-like phenotype (Li et al., 2012; Rockey et al., 2019). Beyond production of abundant amounts of abnormal interstitial collagens (especially type 1 collagen), which significantly alters the ECM composition and increases its stiffness (Kang, 2020; Rockey et al., 2015), one of the most prominent molecular features of activated HSCs is the expression of smooth muscle specific smooth muscle α-actin (SM α-actin or ACTA2), which helps generate a robust actin cytoskeleton (Rockey et al., 2013).

Numerous studies have established that matrix stiffness affects the behavior of HSCs (Friedman, 2008; Martino et al., 2018). For example, when HSCs were cultured on soft substrates that mimic normal liver ECM, they maintained a quiescent phenotype. However, when cultured on stiff substrates that resemble fibrotic livers, they became activated and developed a myofibroblast-like phenotype (Olsen et al., 2011). More importantly, when activated HSCs were grown on soft substrates, they reversed to a quiescent phenotype (Gaca et al., 2003; Olsen et al., 2011; Sohara et al., 2002). These data suggest that liver fibrosis could in theory be reversed if the stimuli to production of the abnormal ECM were interrupted. Indeed, liver fibrosis regression has been demonstrated in animal models (Kisseleva et al., 2012) and in humans (Ellis and Mann, 2012; Rockey and Friedman, 2021). In aggregate, the available evidence suggests that the cellular microenvironment plays a key role in modulating the phenotype switch in HSCs (e.g., activation or quiescence).

### The actin cytoskeleton mediates cell function and mechanotransduction

The actin family consists of 6 highly homologous isoforms, which differ mainly in their amino terminal amino acid sequences (Perrin and Ervasti, 2010). However, each isoform appears to play non-redundant biological roles (Dominguez and Holmes, 2011). Moreover, actin exists either as monomers (globular or G-actin) or filaments (filamentous or F-actin) (Lee and Dominguez, 2010). The switch between G- and F-actin is a highly dynamic molecular process known as actin polymerization and depolymerization (Blanchoin et al., 2014; Heng and Koh, 2010). This process can be monitored by determining the ratio of G/F-actin, an indicator of cytoskeletal actin dynamics (Lee and Dominguez, 2010; Olson and Nordheim, 2010).

In normal liver, quiescent HSCs only express β- and γ-cytoplasmic actin isoforms (i.e., β-actin—ACTB and cyto-γ-actin—ACTG1), and display short and irregular actin filaments in the cytoplasm (Rockey et al., 2019; Shi et al., 2020). In contrast, activated HSCs express abundant SM α-actin, which forms well-organized and robust actin filaments (i.e., stress fibers) (Rockey et al., 2019; Shi et al., 2020). This vigorous and dynamic actin cytoskeleton leads to prominent functional attributes, including increased contractility, proliferation, and cell migration (Rockey et al., 2013; Shi and Rockey, 2017). Of note, the increased expression of SM α-actin appears to additionally play a role in fibrogenesis, as deletion of SM α-actin in HSCs leads not only to decreased cellular contractility, but also to reduced liver fibrosis (Rockey et al., 2019). Similar functional effects of SM α-actin have also been demonstrated in lung, subcutaneous and 3T3 fibroblasts (Hinz et al., 2001; Qin et al., 2018).

Not only is the actin cytoskeleton crucial in cell morphology and function, but it also mediates intracellular mechanotransduction between biophysical and biochemical signals. For instance, we have recently demonstrated that stimulating or targeting cytoskeletal actin dynamics has prominent effects on TGFβ and ET-1 signaling (up- or down-regulation of Smad2/3 and Erk1/2 phosphorylation) in HSCs (Rockey et al., 2019; Shi and Rockey, 2017). The actin cytoskeleton also appears to mediate intracellular Ras-MAPK and NF-kappa B pathways and calcium signaling (Kustermans et al., 2005; Rivas et al., 2004; Smith et al., 2004). Although the relationship between the actin cytoskeleton and mechanotransduction has been well demonstrated, the molecular mechanisms underlying how the actin cytoskeleton informs downstream signaling remain unclear.

### The actin cytoskeleton signals to ECM protein/type 1 collagen expression

Type 1 collagen is a key component of the ECM and its abnormal expression is associated with multiple connective tissue and fibrotic diseases (Arriazu et al., 2014). In liver fibrosis, type 1 collagen is the most abundant component of the abnormal ECM (Arriazu et al., 2014; Kisseleva and Brenner, 2021) and is primarily produced by activated HSCs (Mederacke et al., 2013). The molecular regulation of type 1 collagen is complex (Kisseleva and Brenner, 2021).

The discovery of the upregulation of myocardin and its family member, myocardin-related transcription factor-A (MRTF-A), which have been linked to the actin cytoskeleton-mediated mechanotransduction in activated HSCs, has provided a novel putative mechanism for type 1 collagen regulation during liver injury (Shi et al., 2020; Shi and Rockey, 2017; Shimada and Rajagopalan, 2012). Although myocardin is homologous to MRTF-A in most functional domains, the amino terminus of MRTF-A contains prominent RPEL domains, which form a stable complex with monomeric G-actins, resulting in the cytoplasmic sequestration of MRTF-A (Pipes et al., 2006). While myocardin does not bind actin efficiently, it does form heterodimers with MRTF-A (Pipes et al., 2006). Thus, myocardin is indirectly regulated by actin dynamics. When pro-fibrotic ligands (such as TGFβ and ET-1) activate the Rho signaling pathway (Miao et al., 2002; Peng et al., 2008), actin polymerization occurs, freeing MRTF-A from G-actin and allowing its nuclear translocation and activation of gene transcription (Olson and Nordheim, 2010; Pipes et al., 2006; Shi and Rockey, 2017). Both myocardin and MRTF-A bind serum response factor (SRF—a master transcription factor of actin genes), which binds the CArG boxes of SRF target gene promoters inducing the transcriptional activation of ACTA2 (Olson and Nordheim, 2010; Pipes et al., 2006; Shi et al., 2020; Shi and Rockey, 2017). Of note, myocardin and MRTF-A also mediate Smad2/3 dependent transcriptional activation of COL1A1 and COL1A2 via stimulation of Smad2/3 phosphorylation (Shi and Rockey, 2017). Therefore, in a canonical signaling pathway, myocardin and MRTF-A upregulation leads to the genesis of a robust HSC actin cytoskeleton, which in turns leads to COL1A1 and COL1A2 expression and then increases liver stiffness. Thus, pharmaceutically targeting MRTF-A with the small molecule, CCG-203971, led to decreased fibrosis in the liver (Shi et al., 2020), consistent with other types of tissue wound healing (Sisson et al., 2015).

## Conclusions

**FIGURE 1.**
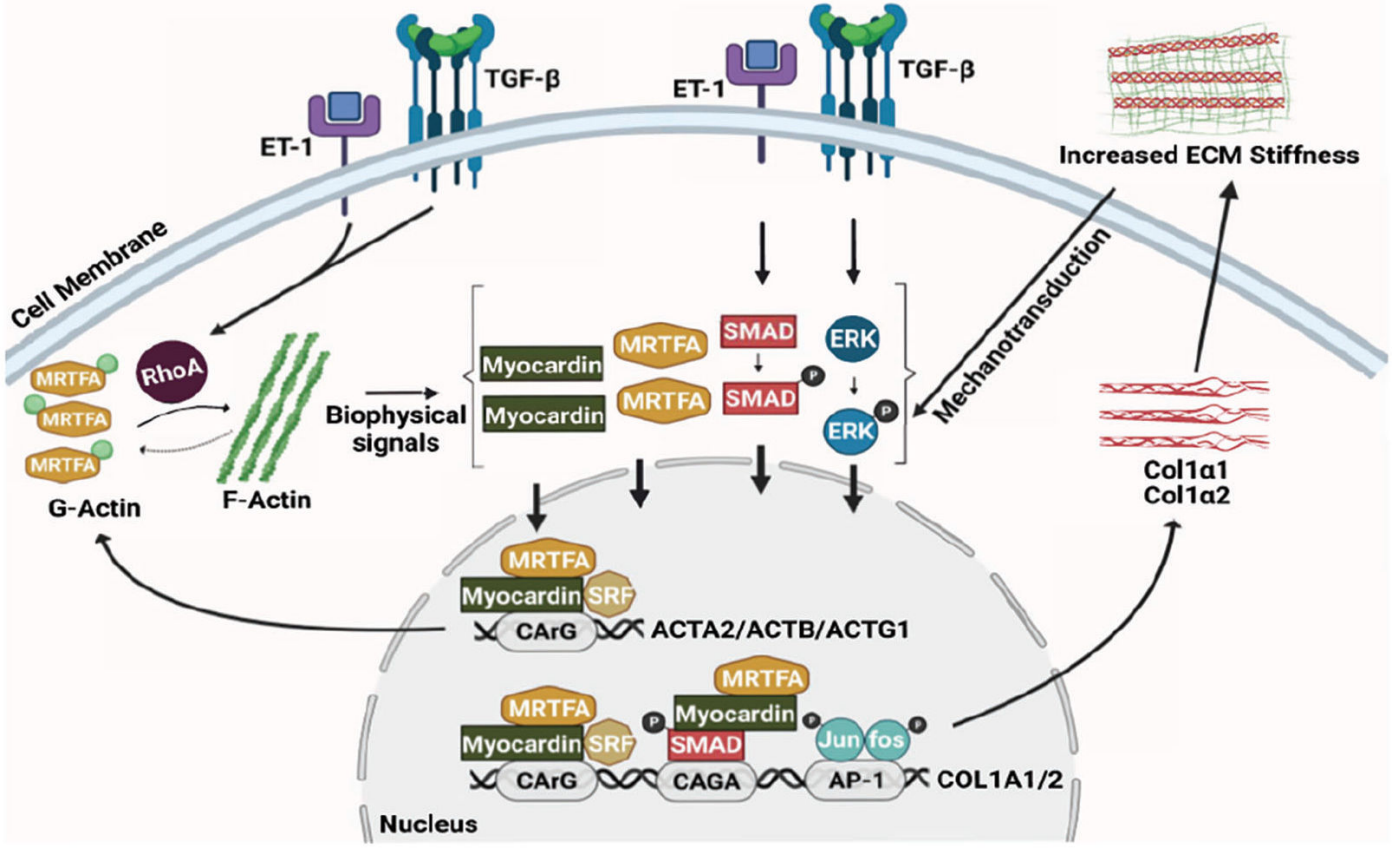
Actin dynamics and stellate cell activation. The schematic diagram shows the interplay between the actin cytoskeleton network and extracellular matrix production in hepatic stellate cells.

The actin cytoskeleton is a highly dynamic structure that continuously polymerizes and depolymerizes as a result of microenvironmental and intracellular signals. Liver injury creates a microenvironment that ultimately alters actin dynamics in HSCs, contributing to their phenotypic transition and abnormal behaviors such as increased proliferation, migration, contraction, and excessive ECM production. Research from our group and others has demonstrated that myocardin and MRTF-A play a key role in the complex regulatory network of actin dynamics and ECM production (Fig. 1). Questions remain—including in particular how the actin cytoskeleton controls downstream signaling to COL1A1 and COL1A2. Deciphering the mechanisms of mechanotransduction could eventually lead to the discovery of novel therapeutic targets and the development of effective drugs to treat liver fibrosis.
